# Identification of Molecular Mechanisms Related to Pig Fatness at the Transcriptome and miRNAome Levels

**DOI:** 10.3390/genes11060600

**Published:** 2020-05-29

**Authors:** Katarzyna Ropka-Molik, Klaudia Pawlina-Tyszko, Kacper Żukowski, Mirosław Tyra, Natalia Derebecka, Joanna Wesoły, Tomasz Szmatoła, Katarzyna Piórkowska

**Affiliations:** 1 Department of Animal Molecular Biology, National Research Institute of Animal Production, Krakowska 1, 32-083 Balice, Poland; klaudia.pawlina@izoo.krakow.pl (K.P.-T.); tomasz.szmatola@izoo.krakow.pl (T.S.); katarzyna.piorkowska@izoo.krakow.pl (K.P.); 2 Department of Cattle Breeding, National Research Institute of Animal Production, Krakowska 1, 32-083 Balice, Poland; kacper.zukowski@izoo.krakow.pl; 3 Department of Pig Breeding, National Research Institute of Animal Production, Krakowska 1, 32-083 Balice, Poland; miroslaw.tyra@izoo.krakow.pl; 4 Laboratory of High Throughput Technologies, Institute of Molecular Biology and Biotechnology, Faculty of Biology, Uniwersytetu Poznanskiego street 6, 61-614 Poznań, Poland; nataliad@amu.edu.pl (N.D.); j.wesoly@amu.edu.pl (J.W.); 5 University Centre of Veterinary Medicine, University of Agriculture in Kraków, Al. Mickiewicza 24/28, 30-059 Kraków, Poland

**Keywords:** pig, fatness, obesity, extracellular matrix, fat deposition, lipid metabolism, NGS

## Abstract

Fat deposition and growth rate are closely related to pork quality and fattening efficiency. The next-generation sequencing (NGS) approach for transcriptome and miRNAome massive parallel sequencing of adipocyte tissue was applied to search for a molecular network related to fat deposition in pigs. Pigs were represented by three breeds (Large White, Pietrain, and Hampshire) that varied in fat content within each breed. The obtained results allowed for the detection of significant enrichment of Gene Ontology (GO) terms and pathways associated directly and indirectly with fat deposition via regulation of fatty acid metabolism, fat cell differentiation, inflammatory response, and extracellular matrix (ECM) organization and disassembly. Moreover, the results showed that adipocyte tissue content strongly affected the expression of leptin and other genes related to a response to excessive feed intake. The findings indicated that modification of genes and miRNAs involved in ECM rearrangements can be essential during fat tissue growth and development in pigs. The identified molecular network within genes and miRNAs that were deregulated depending on the subcutaneous fat level are proposed as candidate factors determining adipogenesis, fatness, and selected fattening characteristics in pigs.

## 1. Introduction

The quantity of fat is a critical factor influencing meat quality and fattening efficiency in pig production. The fat level and mass of adipocyte tissue control food intake and body weight via leptin secretion [1,2]. Leptin is considered an essential hormone regulating adipose tissue metabolism and energy expenditure. Leptin acts as an appetite regulation factor (hunger inhibition and satiety stimulation) through interaction with hypothalamic receptors [3] and controls glucose/lipid metabolism and body weight homeostasis through gluconeogenesis regulation [4]. Moreover, pigs can be a suitable animal model for studies of fat accumulation and obesity [5]. The identification of genetic factors determining fatness levels in pigs can be a valuable resource for medical research in humans.

In humans, genetic predisposition is a key contributing factor in obesity [6]. It has been estimated that the genetic basis of phenotypic variations in obesity can range from 40% to 70% [6,7]. In pigs, the high heritability coefficient (h^2^ = 0.5) indicates the genetic background of fatness traits and the ability to improve them by selection [5]. The identification of genome regions, genes, or single nucleotide polymorphisms (SNPs) related to fatness in pigs has been conducted using different methodological approaches. In 2011, the usage of expression and single nucleotide polymorphism microarrays allowed for the detection of the panel of porcine genes relevant to fatness-associated traits [8]. In 2015, the genome-wide association study (GWAS) method was applied to identify QTLs (quantitative trait loci) located on Sus scrofa chromosome 7 (SSC7) and SSC4 strongly associated with growth and fatness traits based on Chinese pig breeds [9]. Guo et al. [10] using the GWAS method also indicated the involvement of the SSC7 region in the regulation of fatness and growth features. Another approach that can allow for the identification of molecular networks related to fat metabolism and growth traits is transcriptomic research concentrated on examining the hypothalamus–pituitary axis. The hypothalamus is a key brain structure controlling food intake and fat accumulation [11]. The pituitary is a crucial gland involved in fetal adipose metabolism via controlling the fatty acid synthesis and receptor-mediated lipolytic response [12]. Pérez-Montarelo et al. [13] confirmed that the usage of network interaction based on hypothalamic transcriptome analysis could pinpoint candidate genes for fatness in pigs. The transcriptomic profiling of the swine pituitary showed the strong association of this gland with postnatal growth and development [14], and also with fatness-associated traits [15].

A significant number of studies have confirmed an important involvement of adipocyte tissue in food intake [16] and body-weight regulation [17]. Such findings indicated the necessity of the analysis of adipocyte tissue to obtain the full view of metabolic processes related to fatness in pigs. To date, in pigs, transcriptomic research has been applied to identify the regulation processes determining feed efficiency [18] and to examine the body’s response to different nutritional treatments [19]. Processes based on adipocyte tissue gene expression profiling in two Portuguese local pig breeds to determine the fat deposition via controlling of de novo fatty acid synthesis have been proposed, [20]. Furthermore, gene expression profiling in combination with genome resequencing has allowed for the detection of genes potentially related to backfat thickness [21].

The present study aimed to perform comprehensive whole transcriptome and miRNAome analyses [20] of adipocyte tissue to identify the molecular networks strongly related to the most crucial production traits, namely, fatness and feed intake in pigs.

## 2. Materials and Methods

### 2.1. Animals, Phenotype Data Collection, and Tissue Sampling

Animals used for gene and miRNA expression analyses were selected from a large pig population, based on fatness phenotypic traits measured after dissection. All pigs, representing three breeds (Pietrain, Pi; Hampshire, Hp; and Large White, LW) were unrelated (at least three generations back), females, and were maintained under the same housing and feeding conditions according to a procedure previously described [22]. The pigs were kept in individual pens and fed to a weight of 105 kg (±2.5 kg) (based on the Pig Test Station (SKURTCh) of the National Research Institute of Animal Production in Chorzelów). Half-carcasses were dissected 24 h after slaughter and several carcass characteristics, including fatness traits, were evaluated. The traits included average backfat thickness (ABT), calculated from five measurement points (cm) (at the thickest point over the shoulder; on the back above the joint between the last thoracic and first lumbar vertebrae; and at three points over the loin: above the rostral edge (loin I), above the middle (loin II), and the caudal edge (loin III) of gluteal muscle cross-section), and the weight of peritoneal fat (kg), using a procedure described by Tyra and Żak [23].

Pigs for next-generation sequencing (NGS) analysis were selected based on the most important fatness parameter, i.e., ABT. In each analyzed breed (Pietrain, 8, Hampshire, 8, and Large White, 8), pigs with different fatness phenotypes were selected (4 pigs with high and 4 with low fatness). The separation of the two groups with extreme fatness within each breed allowed for avoidance of any potential breed effect on obtained genes or miRNA expression profiles (Table 1). Immediately after slaughter, the samples of fat tissue (subcutaneous fat) were collected into the RNAlater solution (Ambion, Thermo Fisher, USA) and stored at −20 °C.

The research did not require the approval of the Animal Experimentation Committee due to the fact that all biological material was collected from animals which were slaughtered and dissected, and, after carcass evaluation, meat was intended for consumption. The pigs were maintained and slaughtered according to Directive 2010/63/EU of the European Parliament and the Council of 22 September 2010.

### 2.2. Whole Transcriptome Sequencing (RNA-seq)

Extracted total RNA (Direct-zol RNA Mini Prep kit (Zymo Research, Orange, CA, USA)) was subjected to quantity and quality controls using a NanoDrop 2000 spectrophotometer (Thermo Fisher Scientific, Waltham, MA, USA) and a TapeStation 2200 instrument (Agilent, Santa Clara, CA, USA), respectively. Samples with RIN value (RNA integrity number) above 7 were chosen for further analyses. A quantity of 300 ng of total RNA was used for cDNA library preparation according to the TruSeq RNA Kit v2 kit protocol (Illumina, San Diego, CA, USA). The quality and quantity of cDNA libraries were checked using Qubit 2.0 and TapeStation 2200. The individual cDNA libraries were ligated with adaptors with different indexes and pooled. After pooling, each cDNA library was loaded into at least four flow cell lanes as four technical replicates. Sequencing of RNA was performed on the HiScanSQ system (Illumina, San Diego, CA, USA) using the TruSeq SR Cluster Kit v3- CBOT-HS kit and TruSeq SBS Kit v 3 - HS chemistry (Illumina, San Diego, CA, USA) according to the standard protocols and with 81 single-end reads.

The quality of raw data was analyzed using FastQC software (overrepresented sequences, sequence duplication levels, adapter contents, sequence length distribution, sequence quality scores, and GC content). The adapter’s sequence reads shorter than 36 bp and/or reads with a quality score lower than 20 were removed using Flexbar software v2.5 [24]. The estimation of transcript abundance was conducted using RSEM software (v1.2.22) [25] supported by STAR aligner software (STAR_2.4.2a) [26]. The reads were aligned to the Sus scrofa reference genome (assembly Sscrofa11.1, Ensemble release 94). The whole procedure was followed by the alignment parameter evaluation using ENCODE3’s STAR-RSEM pipeline. Differentially-expressed genes (DEGs) were identified according to the following criteria: fold change ≥ |1.0| and adjusted *p*-value < 0.05 separately for each breed using DESeq2 software (version 1.12.4) [26].

### 2.3. MicroRNA Sequencing

MicroRNA libraries were prepared with the use of the NEBNext Multiplex Small RNA Library Prep Set for Illumina (New England Biolabs, Ipswich, MA, USA) according to the protocol. The libraries were barcoded with different indexed primers to allow multiplexing of the samples during NGS sequencing. The amplified libraries were purified and size-selected on a Novex 6% TBE PAGE gel (Invitrogen, Thermo Fisher Scientific, Waltham, MA, USA). The quality and quantity of obtained libraries were measured with a Qubit 2.0 Fluorometer (Thermo Fisher Scientific, USA) and size-assessed with a 2200 TapeStation instrument (Agilent, Santa Clara, CA, USA). The whole miRNA profile sequencing was performed in 36 cycles on a HiScanSQ (Illumina, San Diego, CA, USA) instrument according to the manufacturer’s protocol.

The raw miRNA reads were quality controlled using FastQC software (parameters as for the transcriptome) [27]. The adaptor trimming and filtering were performed with the UEA sRNA Workbench v3.2 helper tool [28], while the miRCat tool v1.0 was used to detect known and novel miRNA sequences. The identification was carried out on the basis of the *Sus scrofa* genome (assembly Sscrofa 10.2) and miRBase v21.0 (Griffiths-Jones lab at the Faculty of Biology, Medicine, and Health, University of Manchester, USA) [29,30], due to the lack of 11.1 assembly in miRbase dataset. The obtained miRNAs genome localization based on 10.2 reference were converted to 11.1 reference using NCBI Genome Remapping tool. The default animal parameters were set except for minimum abundance (6 reads), minimum length (17 nt), and maximum length (25 nt). In the next step, potential other non-coding RNAs were identified and excluded employing the RNAcentral database (the RNAcentral Consortium, 2017). Moreover, isomiR-SEA software (version 1.60) [31] with the default settings was used to identify microRNA length and sequence variants (isomiRs). miRNAs differentially expressed between pigs with low and high fatness in each breed were detected using DESeq2 software (v.1.12.4) [26]. The identification of experimentally-validated target genes and enriched biological processes (KEGG, GO) was performed using the mirPath v.3.0 DIANA Tools web application (DIANA-Lab, Athens, Greece) [32], including TarBase v7.0 (DIANA-Lab, Athens, Greece) as a reference [33]. The analysis was performed based on human homologues deposited in miRBase (21.0) due to the lack of data for pig microRNAs.

### 2.4. Validation of NGS Results

Validation of the RNA-seq results was carried out using a real-time PCR method, for 10 selected DEGs. The primers for selected DEGs were designed based on reference sequences showed in Supplementary Table S1 and using Primer3 Input (version 0.4.0) The detailed information about the chosen genes is presented in Supplementary Table S1. The cDNA was prepared from 300 ng of total RNA using a Maxima First Strand cDNA Synthesis Kit for RT-qPCR (Thermo Fisher Scientific, Waltham, MA, USA) according to the protocol. The exact gene expression levels were measured on a QuantStudio 7 flex instrument (Applied Biosystems, Thermo Fisher Scientific, Waltham, MA, USA) using Sensitive RT HS-PCR EvaGreen Mix (A&A Biotechnology, Gdynia, Poland). The reaction was carried out in three replications for each gene. The expression was calculated using the delta-delta CT method, according to Pfaffl [34] and based on two reference controls *OAZ1* [35] and *RPS29* [36].

Validation of the detected expression levels of 10 selected microRNAs was carried out using the qPCR method. A quantity of 10 ng of total RNA was reverse transcribed to cDNA using a TaqMan Advanced miRNA cDNA Synthesis Kit (Thermo Fisher Scientific, USA) following the protocol. The quantitative estimation of miRNAs was performed on a QuantStudio 7 flex instrument using TaqMan^®^ Advanced miRNA Assays labelled with VIC or FAM fluorescent dye and with TaqMan^®^ Fast Advanced Master Mix (Thermo Fisher Scientific, USA) according to the standard protocol (Supplementary Table S1). The relative expression levels of selected miRNAs were calculated using the delta-delta CT method according to Pfaffl [34] and based on mir-451a as a reference control. The comparison between NGS data (RNA-seq, miRNA-seq) and relative quantity obtained by the real-time PCR method was performed using the Pearson correlation (SAS software, version 8.02).

## 3. Results

### 3.1. RNA Sequencing Results—Differentially-Expressed Genes

The whole transcriptome sequencing of adipocyte tissue allowed for the identification of differentially-expressed genes (DEGs) between pigs varying in backfat thickness. The investigation of three pig breeds enabled the detection of genes related to fatness traits regardless of breed factor. According to the NGS approach, 167 DEGs were identified between Large White pigs with different fatness phenotypes (85 up-regulated and 82 down-regulated genes in pigs with thicker backfat). A total of 32 of these were recognized as novel genes. In turn, in the Pietrain breed, 247 DEGs were detected (53 up-regulated and 194 down-regulated), of which 50 were novel genes or uncharacterized proteins. The highest number of DEGs was identified for the Hampshire breed: 128 up-regulated and 173 down-regulated genes, for a total of 301 genes (46 novel).

**Figure 1:**
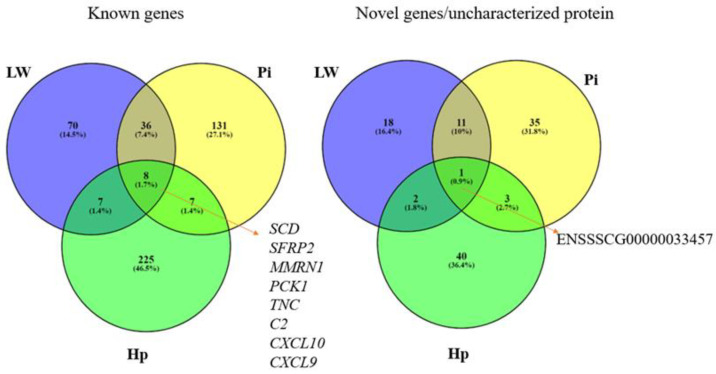
Comparison of differentially-expressed genes (DEGs) between subcutaneous fat tissue varying in thickness in each of three breeds (LW, Large White; Pi, Pietrain; and Hp, Hampshire).

The comparison of the detected sets of DEGs across breeds enabled the identification of eight known genes (*SCD, SFRP2, MMRN1, PCK1, TNC, C2, CXCl10,* and *CXCL9*) and one novel gene common to all three breeds (Figure 1). The largest group of common genes identified as differentially-expressed was found for Large White and Pietrain breeds (44 DEGs and 12 novel genes). The lowest similarity in the identified gene panel was observed for Hampshire pigs and the other two breeds.

**Figure 2:**
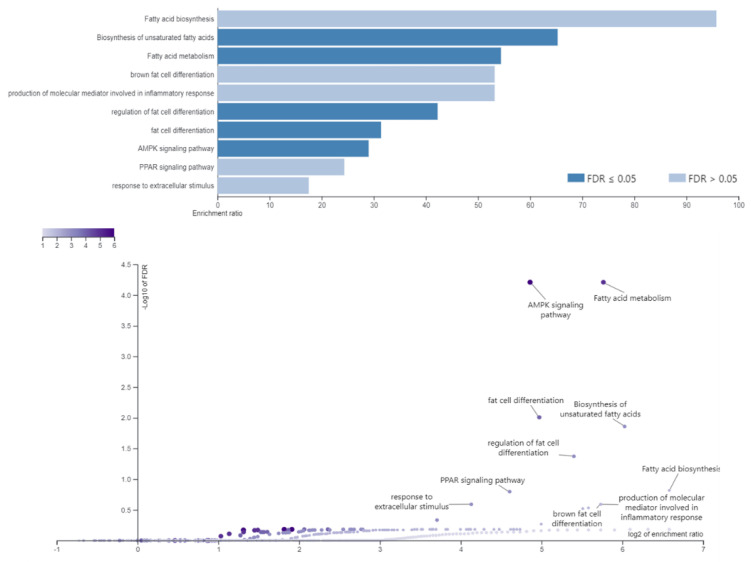
The significant enrichment GO terms and pathways identified based on backfat thickness differences and related to lipid metabolism (WebGestalt software).

**Figure 3:**
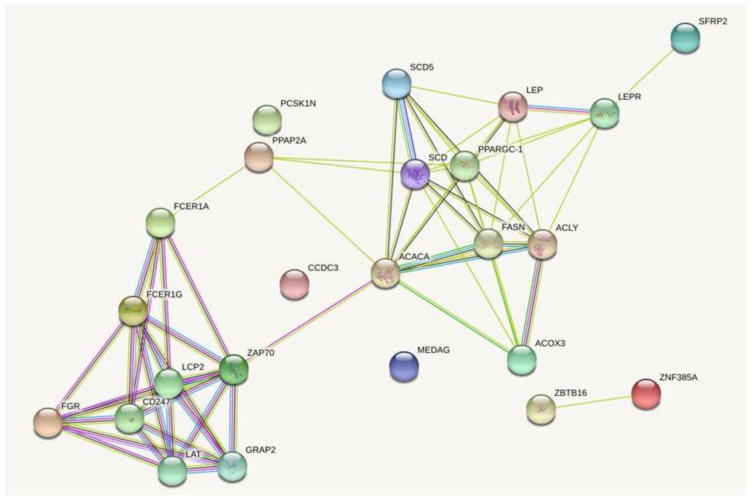
The detected differentially-expressed genes involved in GO terms related to lipid metabolism processes (String software based on Sus scrofa reference; detected genes showed no more than five interactions. Line shape indicates the predicted mode of action: red, interactions that were experimentally determined; blue, interactions from curated databases; black, co-expression; green, text mining associations and interactions based on relevant publications mentioning a transfer from other organisms; yellow, transcriptional regulation).

The Gene Ontology (GO) analysis performed for the DEGs identified in at least two breeds showed significantly-overrepresented GO terms (Table 2). The highest number of genes was assigned to innate immune response (GO:0045087), 14 genes; inflammatory response (GO:0006954), 11 genes; and positive regulation of apoptotic process (GO:0043065), 10 genes. The DEGs were also included in GO terms related to lipid metabolism: fatty acid biosynthetic process, long-chain fatty acid biosynthetic process, positive regulation of fat cell differentiation, positive regulation of mast cell degranulation, and response to excessive food intake (Table 2; David software). Furthermore, GO and pathways analyses using over-representation analysis (WebGestalt software) confirmed that detected DEGs were involved in the biosynthesis of unsaturated fatty acids, fatty acid metabolism, and regulation of fat cell differentiation and brown fat differentiation (not significant GO) (Figure 2). The DEGs associated with several identified GO terms were as follows: *ACACA* (acetyl-CoA Carboxylase α), *SCD* (stearoyl-CoA desaturase), *SCD5* (stearoyl-CoA desaturase 5), *FASN* (fatty acid synthase), *LEP* (leptin), and *CTGF* (connective tissue growth factor) (Table 3; Figure 3).

### 3.2. MiRNA Sequencing Results—Differentially-Expressed miRNAs

**Figure 4:**
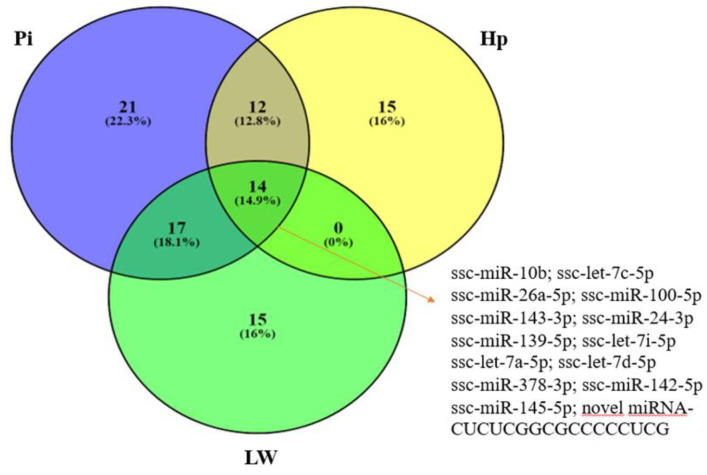
Venn diagram of differentially-expressed miRNAs in subcutaneous fat tissue dependent on fat content in three analyzed breeds (LW, Large White; Pi, Pietrain; and Hp, Hampshire).

The comparison of whole miRNAome profiles between groups of pigs with significant-different fatness traits allowed for the identification of differentially-expressed (DE) miRNAs: 46 for LW (21 up-regulated and 25 down-regulated genes in pigs with thicker backfat), 61 for Pietrain (36 up-regulated and 25 down-regulated), and 41 for Hampshire (8 up-regulated and 31 down-regulated). As for DEGs, analogous comparison across breeds was also carried out for DE miRNAs and showed the presence of 14 miRNAs common to all three breeds (Figure 4). The lowest number of common miRNAs was detected for Hampshire and Large White pigs. Detailed data on the chromosomal localization of the identified miRNAs according to Sscrofa10.2 and Sscrofa11.1 assemblies are in the Supplementary Table S2.

**Figure 5:**
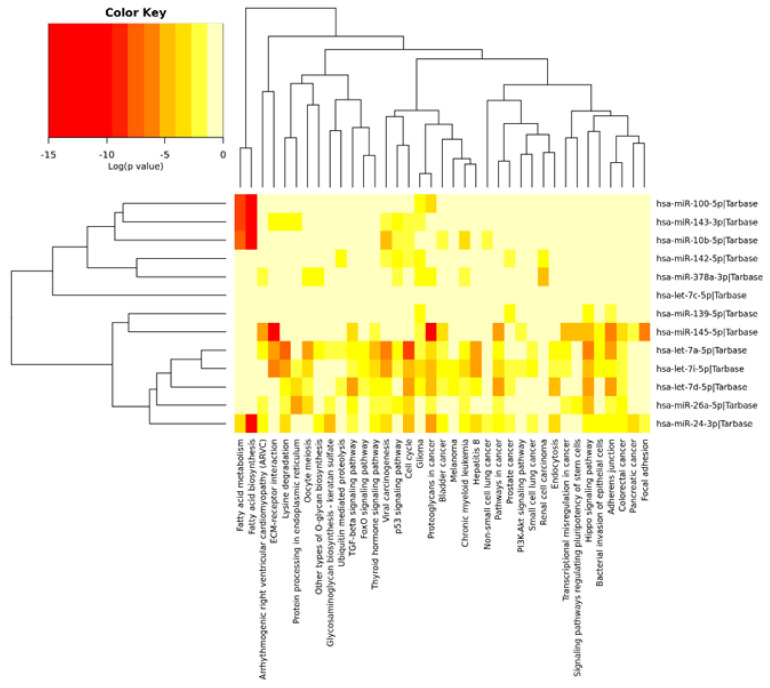
Venn diagram of differentially-expressed miRNAs in subcutaneous fat tissue dependent on fat content in three analyzed breeds (LW, Large White; Pi, Pietrain; and Hp, Hampshire).

The GO analysis performed using the mirPath v.3 DIANA tool showed that differentially-expressed miRNAs were involved in extracellular matrix organization (GO:0030198), extracellular matrix disassembly (GO:0022617), and innate immune response (GO:0045087), where the highest number of predicted targeted genes for miRNAs was detected. Eight miRNAs were associated with cellular lipid metabolic process (GO:0044255) (Table 4). Moreover, miR-100-5p, miR-143-3p, miR-10b-5p, and miR-24-3p were involved in fatty acid metabolism and fatty acid biosynthesis (Figure 5). The predicted targeted genes for differentially-expressed miRNAs are summarized in Supplementary Table S3.

### 3.3. Analysis of Pathways Common for DEGs and DE miRNAs

The pathways analysis was performed for both DEGs and DE miRNAs identified in at least two breeds. Furthermore, the predicted genes regulated by the identified miRNAs, and involved in selected pathways, detected using mirPath v.3 DIANA tool are shown in Table 5.

An enriched pathway significant for both DEGs and DE miRNAs was fatty acid metabolism (KEGG ID: hsa01212 for miRNA/ ssc01212 for DEGs) for which four miRNAs and five genes were detected. Three of all DEGs involved in fatty acid metabolism overlapped with predicted genes regulated by detection miRNAs, namely: *SCD, FASN,* and *ACACA*. A similar situation was observed for the fatty acid biosynthesis pathway (*p*-value for miRNAs < 1.0 × 10^−325^; not significant for DEGs); *FASN* and *ACACA* differentially-expressed genes overlapped with miRNA-predicted targets. The pathways identified as significant and common for DE miRNAs and DEGs were also the Hippo-signaling pathway, ECM−receptor interaction, cell cycle, and P53-signaling pathways.

The pathway analysis of DE miRNAs also showed significant enrichment for the set of pathways related to toll-like receptors (toll-like receptor 2, 3, 4, 5, 9, and 10 signaling pathways; toll-like receptor TLR1:TLR2 signaling pathways; and toll-like receptor TLR6:TLR2 signaling pathways), as well as TRIF-dependent toll-like receptor signaling pathways (Figure S1).

### 3.4. Validation of Obtained Data

The validation of RNA-seq results was performed for eight DEGs and six miRNAs (Table 6). For the majority of analyzed DEGs, the RNA-seq data and RQ results showed high and significant correlation coefficients (from 0.66 to 0.95), which confirmed the reliability of the NGS results. The highest similarity of results was obtained for *PCK1, ACACA*, and *LEP* genes. For four miRNAs, high correlation coefficients were detected, but these were significant only for miR-26a-5p, mir-100-5p, and mir-103a-3p.

## 4. Discussion

In pigs, fatness traits are one of the important production features due to their strong relationship with meat quality and fattening performance. The inverse correlation between lean meat content and fatness traits results in the decrease of the fatness level in carcasses as meatiness increases [37]. Moreover, the proper fat content, including of intramuscular fat (IMF), is critical to achieving the desired meat quality parameters. The adipocyte tissue, as a secretory organ, also determines the regulation of food intake; thus, the body fat content can influence food intake and body weight [2]. In turn, the food intake and feed conversion ratio (FCR) are primary factors which determine the economic efficiency of pig production.

The high phenotypic variability of fatness characteristics, as well as the heritability coefficient (about 0.5), strongly indicate the genetic background of this group of traits and the possibility of their improvement or modification using genetic markers [5]. From a breeding perspective, the most important step would be to establish such a genetic marker that enables the controlling of pig fatness without losing the already-achieved level of meatiness. The latest successes of high throughput genetic methods, such as NGS sequencing technology and the GWAS approach, have become more applicable and create new possibilities in the search for and identification of the molecular background of phenotypic variations.

In the presented report, comprehensive whole mRNA and miRNA profiling of adipocyte tissue was applied to detect the molecular network related to fat deposition in pigs. The use of three pig breeds representing different usage types (maternal line, Large White; sire lines, Pietrain and Hampshire) allowed indication of a universal genetic basis of fatness characteristics that was not associated with any breed. The transcriptome sequencing of fat tissue delivered from pigs diverse in terms of backfat thickness enabled the pinpointing of significant, enriched Gene Ontology terms and pathways possibly related to fat deposition. For both DEGs and DE miRNAs, the fatty acid metabolism pathway was detected as significant. This pathway involved three DEGs, namely, *ACACA, FASN*, and *SCD,* and differentially-expressed miRNAs that recognized these genes as their targets. Moreover, the stearoyl-CoA desaturase gene (*SCD*) was identified as significant for all three analyzed breeds, and the *SCD* expression level was higher in pig groups with thicker fat cover. The stearoyl-CoA desaturase plays a vital role during the biosynthesis of monounsaturated fatty acids, within palmitoyl- and oleoyl-CoA, making them readily available to the body [38]. The higher *SCD* expression observed in this study, in pigs with higher fat content was in accord with previously-found human data. Hulver et al. [39] showed that an increased *SCD* level is correlated to obesity. Similarly, decrease of *SCD* transcription leads to a reduction of adiposity [40]. The deficiency of stearoyl-CoA desaturase results in leanness and leads to an increase of metabolic rate as well as insulin sensitivity [38]. Moreover, a study performed on ob/ob mice indicated that *SCD* is involved in metabolic response to leptin and down-regulation of *SCD* can be a key element of the metabolic actions of leptin [41].

Reports of many authors have indicated the association of the *SCD* gene and selected fatness traits in pigs. Using the GWAS approach, Yang et al. [42] showed a possible relationship between the *SCD* locus and C18:0 content. Similarly, results from the expression Genome-Wide Association Study (eGWAS) indicated that the *SCD* gene is related to intramuscular fat composition in pigs [43]. Xing et al. [21] compared transcriptomes of subcutaneous adipose tissue of Landrace pigs depending on variable backfat levels, which showed significantly-higher *SCD* expression in animals with increased fatness. The authors proposed stearoyl-CoA desaturase as a candidate gene for fat deposition. Our results, based on three different pig breeds, confirmed previous observations, thus strongly supporting an essential role of the *SCD* gene in the determination of fat-associated traits.

Moreover, the present miRNAome sequencing identified that expression of miR-24-3p is also dependent on subcutaneous fat level. In turn, the mirPath v.3 and TarBase v7.0 DIANA bioinformatic tools found that this microRNA modulates expression of the *SCD* gene, as well as fatty acid synthase (*FASN*), acetyl-CoA carboxylase α (*ACACA)*, acetyl-coA acyltransferase 1 and 2 (*ACAA1* and *ACAA2*), and malonyl coA-acyl carrier protein transacylase (*MCAT*). miR-24-3p has been established to play a role in adipogenesis via activating adipocyte differentiation by targeting genes engaged in MAPK7-signaling pathways [44,45]. To date, only a few reports have been published concerning the possible involvement of miR-24-3p in lipid metabolism processes and obesity development. The present study indicates that this miRNA can be related to the regulation of fat deposition processes by targeting the key genes of fatty acid metabolism.

The analogues gene expression profile—increased transcript level in all fatty pigs independent of breed—was also observed for *ACACA* and *FASN* genes. A recent study applied the RNA-seq method to analyze the whole transcriptome profile of subcutaneous fat in native Puławska pigs that differed in backfat tissue [46]. The authors identified the higher transcript level of all three genes—*ACACA, FASN*, and SCD—in pigs with increased backfat thickness. Previous research showed the significant association of the *FASN* gene with meat quality and the *ACACA* gene with IMF content. In Iberian pigs, the *ACACA* gene was proposed as a candidate gene responsible for intramuscular content of saturated fatty acids and monounsaturated fatty acids fatty acids [47]. Furthermore, the up-regulation of *FASN* and *SCD* genes were reported in Alentejano pigs and related to the higher fat deposition observed in this breed [20]. The research performed on Iberian × Landrace pig cross lines indicated a significant association of the *ACACA* gene and IMF palmitic fatty acid percentage [48]. Similarly, the *FASN* gene was previously correlated to meat quality, fatty acid content, and composition [49,50]. Our research, supported by previous studies, strongly confirms that the identified DEGs determine fat deposition in pigs.

### Enriched Metabolic Process and Pathways Associated with Fat Deposition

Genes whose expression varied between thick and thin backfat groups belonged to several processes and pathways directly related to fatty lipid metabolism, within the fatty acid biosynthetic process, the long-chain fatty acid biosynthetic process, positive regulation of fat cell differentiation, and response to dietary excess. The molecular network of food intake controlled by adipocyte tissues has been widely investigated and described. One of the main factors determining the energy balance by hunger inhibition is leptin [51]. The DEGs set comparison showed that *LEP* gene expression was increased in fat tissue of more obese pigs independent of breed. These findings are in accordance with observations made in humans, where the overexpression of the leptin gene in subcutaneous and omental adipose tissues in obese patients was detected [52]. Adipocyte cells accumulate triglyceride and increase the synthesis of leptin during their growth. Leptin acts via the hypothalamus to control energy balance and feeding behavior [53] Thus, leptin, also called an anti-obesity hormone, reduces food intake and increases energy expenditure [54]. Likewise, the other two DEGs identified in the present study—*PPARGC1A* (peroxisome proliferator-activated receptor-γ co-activator-1alpha) and *PCSK1N* (proprotein convertase subtilisin/kexin type 1 inhibitor)—have been associated with obesity in humans. A polymorphism in the *PPARGC1A* gene is related to obesity and type 2 diabetes [55], while the down-regulation of *PCSK1N* gene expression is also associated with obesity [56]. In the present research, *PPARGC1A* expression was up-regulated in thicker backfat tissue in all three investigated pig breeds, whereas expression of the *PCSK1N* gene was lowered in obese pigs represented by Hampshire and Large White breeds. This suggests that all identified DEGs related to mast cell (MC) degranulation can affect the increase of fat tissue mass and lipid metabolism.

Research performed in humans and animals strongly indicates that MCs are involved in activation of adipocytes and recruitment of inflammatory cells [57]. The positive regulation of the MC degranulation-GO term was identified as significantly deregulated in fat tissue dependent on fat cover thickness. The research allowed for the detection of *FGR, FCER1A, FCER1G,* and *ZAP70* as DEGs associated with MC degranulation. The function of MC degranulation is associated with the release of multiple enzymes which can influence adipocyte tissue modification. In vitro studies on human white adipocyte tissue showed that MCs can promote adipose initiation in response to cold [58]. Furthermore, MC cells induced remodeling of adipose tissue extracellular matrix [57].

Interestingly, the obtained data showed the significant enrichment of ECM organization (GO:0030198) and ECM−receptor interaction pathways. The panel of DEGs was identified including genes coding for collagens, thrombospondins, and laminin, which belong to extracellular matrix remodeling. A recent report highlighted that ECM plays a critical role in adipose tissue expansion through controlling cell migration during body growth and development [59]. It has been proven that adipose tissue expansion is strongly related to ECM remodeling at the level of matrix individual components—collagens, thrombospondins, metalloproteinases, and their inhibitors (Tissue Inhibitor of Metalloproteinase - TIMPs) [60]. The present research allowed the identification of DE miR-145-5p and its target gene, tenascin C (*TNC*), both of which are involved in the ECM−receptor interaction pathway. Tenascin C, as a glycoprotein member of ECM, is related to tissue developmental processes, while miR-145 is involved in abdominal obesity, insulin resistance, and lipid metabolism [61]. The exact role of miR-145 has not been well established but the increased expression of miR-145 was observed in adipose and liver tissues in obese patients [62]. It has been proposed that this miRNA plays a role in lipolysis and our results supports this thesis, and also confirm that miR-145 overexpression in fat tissue is associated with obese individuals, which was independently observed in the investigated pig breeds.

Another interesting observation is the down-regulation of the *TNC* gene identified in pigs with thicker fat cover. Tenascin C determines the formation of the collagen network in adipocyte tissue controlling cell migration and proliferation [63]. On the other hand, the excessive cross-linking of adipocytes by fibrotic components can lead to the reduction of their metabolic activity [64]. We hypothesized that the increased expression of the *TNC* gene in thinner backfat can be related to significant modification of extracellular matrix components, which may result in reduced adipocyte activity and a decreased rate of fat tissue development. Tenascin C is also related to activation of the toll-like receptor 4 (TLR4) signaling pathway, which triggers the obesity-induced inflammatory response [65]. Our results also showed the significant enrichment of both toll-like receptor TLR1:TLR2 signaling and toll-like receptor TLR6:TLR2 signaling pathways for DE miRNAs. Gene expression profiling of human peripheral blood mononuclear cells (PBMCs) showed the increased transcript level of *TLR2* and *TLR4* genes in obese patients [66]. The increase in expression of the *TLR4* gene, reported in differentiating adipocytes in db/db mice suggests that this gene is critical during obesity development processes [67]. In the present study, in addition to the detection of GO terms related to *TLR2* and *TLR6*, the set of DE miRNAs targeting the *TLR4* gene was identified, namely, the members of the let-7 family (let-7a-5p, let-7i-5p, and let-7d-5p). To date, many reports have indicated a significant role of let-7 miRNAs in regulation of adipogenesis, lipolysis, and insulin resistance [68]. The differential expression of several let-7 family members may confirm that, beside *TLR* genes, this miRNA family plays a key role in the fat deposition rate and lipid metabolism.

The other proven role of ECM receptor pathways during fat tissue development is the regulation of adipocyte apoptosis and/or necrosis processes [60]. The results obtained in our study also indicated expression changes of genes involved in the positive regulation of apoptotic process GO term and cell cycle pathways. Other members of the EC matrix—thrombospondins—such as those identified in our study (*THBS2, THBS3,* and *THBS4*), are mainly detected in visceral adipose tissues and their increased expression level is associated with obesity. Moreover, thrombospondin 1 has been proposed as a novel marker related to obesity and metabolic syndrome [69]. The identification of the panel of genes involved in ECM receptor pathways and extracellular matrix assembly confirmed that such signaling is essential for fat deposition processes in pigs irrespective of a breed factor.

## 5. Conclusions

The backfat thickness and growth rate are closely related to pork quality and fattening efficiency, as well as reproductive and immune characteristics. Taking into account whole transcriptome profiling of pigs varying in fat content across three pig breeds, the significant enrichment of Gene Ontology terms and pathways associated directly and indirectly with fat deposition was detected (*ACACA, ACOX3, FASN, SCD5, SCD,* and *PLPP1).* The differentially-expressed miRNAs and genes were involved in fatty acid metabolism, positive regulation of fat cell differentiation, and the inflammatory response. Moreover, the results showed that adipocyte tissue content regulated the expression of leptin and other genes related to a response to dietary excess. Our results confirmed previous findings in humans that showed ECM organization and disassembly are fundamental for fat tissue growth and development. The modification of genes and miRNAs involved in ECM rearrangements can also be essential during fat tissue growth and development in pigs. We also suggest that mast cell degranulation can be one of the significant processes associated with adipocyte tissue development. The pinpointed molecular networks within genes and miRNAs deregulated by subcutaneous fat level are proposed as candidate factors determining adipogenesis, fatness, and selected fattening characteristics in pigs. Identified DEGs and DE miRNAs should be investigated in the future in terms of their use as genetic markers associated with pig production traits.

## Figures and Tables

**Table 1:** The differences in fatness traits detected in all three breeds and obtained pig groups used in genetic analyses.

	Backfat Thickness (cm)				Weight of Peritoneal Fat (kg)			
	L		H		L		H	
Pietrain	0.71	±0.0.1 ^b^	1.29	±0.10 ^a^	0.15	±0.001 ^b^	0.31	±0.051 ^a^
Hampshire	0.99	±0.03 ^b^	1.54	±0.20 ^a^	0.30	±0.016	0.29	±0.067
Large White	1.04	±0.06	1.40	±0.14	0.23	±0.070	0.31	±0.122

L, low fatness; H, high fatness. Data are presented as means ± standard error; means with different letters (a,b differ significantly, *p*-value = 0.05).

**Table 2:** Significant, enriched Gene Ontology terms for differentially-expressed genes (DEGs) in relation to backfat thickness.

Gene Ontology/Accession Number	FDR *	N	Gene Name
Extracellular matrix organization (GO:0030198)	9.2 × 10^−3^	6	*CYR61; ELN; GFAP; HPSE; POSTN; VTN*
Positive regulation of mast cell degranulation (GO:0043306)	2.4 × 10^−3^	4	*FGR; FCER1A; FCER1G; ZAP70*
Cell adhesion (GO:0007155)	3.5 × 10^−3^	12	*TNFAIP6; WISP1; CTGF; CYR61; HAS1; LYVE1;* *NOV; POSTN; SELL; TNC; THBS2; THBS3*
Innate immune response (GO:0045087)	5.9 × 10^−2^	14	*FGR; FCER1G; MX1; MX2; S100A8; B2M; BST2; FGB; IFIH1; JCHAIN; LCN2; PML; TLR2; ZAP70;*
Fatty acid biosynthetic process (GO:0006633)	8.4 × 10^−3^	4	*ACACA; SCD; FASN* *; ACLY*
Positive regulation of apoptotic process (GO:0043065)	5.6 × 10^−3^	10	*BMF; ALDH1A2; CLU; CYP1B1; GADD45G; IGFBP3; SFRP2; TOP2A; TGM2; ZBTB16*
Long-chain fatty acid biosynthetic process (GO:0042759)	6.0 × 10^−3^	3	*PLPP1; SCD; SCD5*
Response to dietary excess (GO:0002021)	8.6 × 10^−3^	3	*PPARGC1A; LEP; PCSK1N*
Positive regulation of fat cell differentiation (GO:0045600)	6.8 × 10^−3^	5	*CCDC3; MEDAG; SFRP2; ZBTB16; ZNF385A*
Inflammatory response (GO:0006954)	8.6 × 10^−3^	11	*CCL5; CCR5; CD180; FAS; CXCL10; C5AR1; PLP1; TSPAN2; TBXA2R; TLR2; ZAP70*

* FDR (false discovery rate); *p*-values are shown after Benjamini correction and according to David software; N, number of identified DEGs.−

**Table 3:** The fold-change values of DEGs related to lipid metabolism and detected as significant for at least two breeds.

Gene	Accession Number	Pietrain		Large White		Hampshire	
		**FC**	**FDR**	**FC**	**FDR**	**FC**	**FDR**
*LEP*	ENSSSCG00000040464	1.41	0.03	2.14	0.001	1.10	ns
*ACACA*	ENSSSCG00000017694	1.62	0.03	1.67	0.001	1.52	ns
*SCD*	ENSSSCG00000010554	2.95	0.001	1.66	0.001	2.23	0.04
*SCD5*	ENSSSCG00000009245	−1.32	0.04	−1.09	ns	−2.28	0.001
*FASN*	ENSSSCG00000029944	1.58	ns	1.47	0.02	3.07	0.005
*ACOX3*	ENSSSCG00000008724	1.40	0.05	1.32	ns	2.06	0.001
*C2*	ENSSSCG00000001422	−3.18	0.001	−2.00	0.001	2.31	0.01
*ACLY*	ENSSSCG00000017421	1.65	0.05	2.83	0.001	1.13	ns
*TNC*	ENSSSCG00000005494	−3.23	0.0004	−2.55	0.001	−4.17	ns
*PPARGC1A*	ENSSSCG00000029275	1.16	ns	13.45	0.0001	3.27	0.05
*PCSK1N*	ENSSSCG00000021328	1.07	0.05	−1.52	ns	−2.99	0.05
*TLR2*	ENSSSCG00000009002	−2.63	0.001	−1.59	0.05	−1.34	ns
*FGR*	ENSSSCG00000003578	1.13	0.05	1.62	0.001	−1.10	ns
*FCER1A*	ENSSSCG00000006413	1.40	0.001	−1.19	0.01	−2.69	ns
*FCER1G*	ENSSSCG00000006357	−2.40	0.001	−1.40	0.05	−1.22	ns

FDR (false discovery rate); FC, fold change; ns, not significant.

**Table 4:** Significant, enriched Gene Ontology terms for differentially-expressed miRNAs in relation to backfat thickness.

Gene Ontology/Accession Number	*FDR* *	N Target Genes	N miRNAs	miRNAs
Extracellular matrix organization (GO:0030198)	<1.0 × 10^−325^	113	5	hsa-let-7a-5p; hsa-let-7i-5p; hsa-miR-24-3p; hsa-miR-145-5p; hsa-let-7d-5p
Extracellular matrix disassembly (GO:0022617)	<1.0 × 10^−325^	46	6	hsa-let-7a-5p; hsa-let-7i-5p; hsa-miR-24-3p; has-miR-26a-5p; hsa-miR-145-5p; hsa-let-7d-5p
Cellular lipid metabolic process (GO:0044255)	3.1 × 10^−13^	63	8	hsa-let-7a-5p; hsa-let-7i-5p; hsa-miR-24-3p; has-miR-26a-5p; has-miR-143-3p; has-miR-142-5p; has-miR-145-5p; hsa-let-7d-5p
Cell junction organization (GO:0034330)	<1.0 × 10^−325^	66	6	hsa-let-7a-5p; hsa-let-7i-5p; hsa-miR-24-3p; has-miR-143-3p; hsa-miR-145-5p; hsa-let-7d-5p
MyD88-independent toll-like receptor signaling pathway (GO:0002756)	<1.0 × 10^−325^	44	7	hsa-let-7a-5p; hsa-let-7i-5p; hsa-miR-24-3p; has-miR-26a-5p; hsa-miR-145-5p; hsa-let-7d-5p; has-miR-378a-3p
Cellular component disassembly involved in execution phase of apoptosis (GO:0006921)	<1.0 × 10^−325^	28	7	hsa-let-7a-5p; hsa-let-7i-5p; hsa-miR-24-3p; has-miR-26a-5p; hsa-miR-145-5p; hsa-miR-145-5p; hsa-miR-378a-3p
Toll-like receptor TLR1:TLR2 signaling pathway (GO:0038123)Toll-like receptor TLR6:TLR2 signaling pathway (GO:0038124)	<1.0 × 10^−325^	39	7	hsa-let-7a-5p; hsa-let-7i-5p; hsa-miR-24-3p; has-miR-26a-5p; hsa-miR-139-5p; has-miR-378a-3p; hsa-let-7d-5p
Innate immune response (GO:0045087)	<1.0 × 10^−325^	213	7	hsa-let-7a-5p; hsa-let-7i-5p; hsa-miR-24-3p; has-miR-26a-5p; hsa-miR-139-5p; has-miR-142-5p; has-miR-145-5p; hsa-let-7d-5p

* *p*-values are presented after FDR correction and according to the mirPath v.3 tool; N target genes, the number of predicted target genes of differentially-expressed miRNAs; N miRNAs, number of DE miRNAs.

**Table 5:** Significant pathways detected for both DEGs (significant for at least two breeds) and DE miRNAs (14 miRNAs common for all breeds).

	DE miRNAs					DEGs		
Pathways	FDR *	N	miRNAs	N	N Target Genes	FDR*	N	Genes
Fatty acid metabolism (hsa01212/ssc01212)	6.6 × 10^−16^	4	hsa-miR-100-5p; hsa-miR-10b-5p; hsa-miR-143-3p; hsa-miR-24-3p	9	*FASN; ACACA;* *MCAT; ACAA2; ACAA1; CPT1A; CPT2; SCD; ELOVL5*	5.4 × 10^−3^	5	*ACACA;* *ACOX3; FASN; SCD5; SCD*
ECM−receptor interaction (hsa04512/ssc04512)	<1.0 × 10^−325^	4	hsa-let-7a-5p; hsa-let-7i-5p; hsa-miR-143-3p; hsa-miR-145-5p	35	*SPP1; CD44; LAMC1; LAMC3; ITGA3; ITGA5; FN1; TNC; COL1A2; COL4A2; COL5A1; COL5A2; COL6A1; COL27A1; ITGB1; GP5; THBS1*	2.4 × 10^−2^	11	*CHAD; COL1A1; COL5A3; COL6A6; COL11A1; LAMC2; TNC; THBS2; THBS3; THBS4; VTN*
Hippo-signaling pathway (hsa04390/ssc04390)	<1.0 × 10^−325^	7	hsa-let-7a-5p; hsa-let-7i-5p; hsa-miR-145-5p; hsa-miR-24-3p; hsa-miR-139-5p; hsa-let-7d-5p; hsa-miR-26a-5p	87	*PPP2CA; BMP5; BMP2; BMP7; FGF1; ACTG1; PPP1CB; CTGF;* Figure S2	0.01	7	*YWHAH; CTGF; FZD2; FZD4; NKD1; TGFB3; WNT2B*
Fatty acid biosynthesis (hsa00061/ssc00061)	<1.0 × 10^−325^	4	hsa-miR-100-5p; hsa-miR-10b-5p; hsa-miR-143-3p; hsa-miR-24-3p	3	*FASN; ACACA* *; MCAT*	ns	2	*FASN; ACACA*
Cell cycle (hsa04110/ssc04110)	<1.0 × 10^−325^	9	hsa-let-7a-5p; hsa-let-7i-5p; hsa-miR-10b-5p; hsa-miR-143-3p; hsa-miR-24-3p; hsa-miR-26a-5p; hsa-miR-142-5p; hsa-let-7d-5p	78	*ESPL1; CDC6; GSK3B; CCNB2; RBL2; PCNA; E2F1* *E2F2;* *YWHAE; MCM6; MCM2* Figure S3	0.003	5	*E2F2* *; GADD45G; MCM2;* *TGFB3; YWHAH;*
P53-signaling pathways (hsa04115/ ssc04151)	2.5 × 10^−9^	7	hsa-let-7a-5p; hsa-let-7i-5p; hsa-miR-26a-5p; hsa-miR-143-3p; hsa-miR-10b-5p; hsa-miR-142-5p; hsa-miR-378-3p	60	*ZMAT3; CCNB2; CCNB1;* *CDK4; BID; THBS1; CDK2* *CCND2; PERP; RRM2B; CDK1; CDKN2A; CDK6* *CHEK1; TP53; APAF1; PMAIP1; CD82; CASP3*	0.0002	13	*CHAD; COL1A1; COL6A6; LAMC2; PCK1; THBS2; THBS3; THBS4; TLR2; TNC; VTN; YWHAH*

Accession numbers are presented for DE miRNAs/DEGs from the mirPath v.3 and KEGG database, respectively; * *p*-values are presented after FRD correction and according to the mirPath v.3 tool for DE miRNAs and after Benjamini correction and according to David software for DEGs; N, number of identified DEGs; ns, not significant; highlighted genes were identified both as differentially-expressed and as predicted genes regulated by miRNAs. The bold and underline were used to highlighted the genes identified in both–DEGs and targeted genes groups.

**Table 6:** Correlation coefficients for NGS results and qPCR data for both miRNAs and DEGs.

DEGs	Correlation	miRNAs	Correlation
*ROCK1*	0.56	hsa-miR-26a-5p	0.81 *
*LRP12*	0.66 *	hsa-let-7a-5p	0.50
*ACACA*	0.91 **	hsa-mir-100-5p	0.75 *
*HK2*	0.87 **	hsa-mir-378a-3p	0.40
*LRP6*	0.75 *	hsa-mir-103a-3p	0.88 *
*LEP*	0.94 **	hsa-miR-125b-5p	0.73
*TNC*	0.83 *		
*PCK1*	0.95 **		

* *p*-value < 0.05; ** *p* < 0.01.

## Supplementary Materials

The following are available online at https://www.mdpi.com/2073-4425/11/6/600/s1, Table S1. Detailed information about DEGs and miRNAs whose expression was estimated using real-time PCR methods. Table S2. Detailed data on the chromosomal localization of the identified miRNAs according to Sscrofa10.2 and Sscrofa11.1 (“LW” stands for Large White samples, “Pi” denotes Pietrain samples, and “Hp” stands for Hampshire samples; “NA” denotes potentially novel miRNA identified in this study). Table S3. TarBase experimentally supported interactions—the predicted targeted genes (N) for each of the 14 detected miRNAs. Figure S1. The significant enrichment molecular pathways in which 14 microRNAs commonly detected in all three pig breeds were detected (DIANA-miRPath v3.0, DIANA-Lab, Athens, Greece). Figure S2. The significant enrichment Hippo-signaling pathway (KEGG database, hsa04390) involved genes targeted for identified differentially-expressed miRNAs. Targeted genes are marked orange (genes included in more than one pathway) and yellow (genes included only in one pathway). Figure S3. The significant enrichment cell cycle (KEGG database; hsa04110) involved genes targeted for identified differentially-expressed miRNAs. Targeted genes are marked orange (genes included in more than one pathway) and yellow (genes included only in one pathway).
