# Supplementary material for: Identification of Molecular Mechanisms Related to Pig Fatness at the Transcriptome and miRNAome Levels
Source: Genes (Basel). 2020 May 29;11(6):600. doi: 10.3390/genes11060600 (PMC7348756; doi:10.3390/genes11060600)
Supplement: Supplementary file 1 [file genes-11-00600-s001.zip › Table S1.docx]

| Table S1. The detail information about DEGs and miRNAs which expression was estimated using real-time PCR methods. | | | | |
| --- | --- | --- | --- | --- |
| DEGs |  |  |  |  |
| Gene | Gene name | Accession numer | Primers | Amplicon lenght [bp] |
| *ROCK1* | Rho Associated Coiled-Coil Containing Protein Kinase 1 | ENSSSCG00000021893 | F CCTGCCCTAGAATGTCGAAG  R AGAAAGTGTTCGAGGGGATG | 231 |
| *LRP12* | LDL receptor related protein 12 | ENSSSCG00000006039 | F GTGGAAATGGCAAGTGTGTG  R CTTTGGTAAACCGGGACAGA1 | 168 |
| *ACACA* | acetyl-CoA carboxylase alpha | ENSSSCG00000017694 | F CAAGGTCGAGACCGAAAGAA  R GGGTGTGACCATGACAACAA | 219 |
| *HK2* | Hexokinase 2 | ENSSSCG00000008261 | F GCCGCTCTCATCCTTTACAC  R GGTAGCTCCAAGCCCTTTCT | 230 |
| *LRP6* | LDL receptor related protein 6 | ENSSSCG00000000625 | F TATCAGTGTGCTTGCCCAAC  R ATGGCCCTCACTTCATCATC | 236 |
| *LEP* | leptin | ENSSSCG00000040464 | F GCTCGCGCGTCTATAAGAG  R ACGGCTTCAACGTAGGACAG | 171 |
| *TNC* | tenascin C | ENSSSCG00000005494 | F GCGTCTGCTTTGAAGGCTAC  R CACACGCACTCATTCTCCAC | 196 |
| *PCK1* | phosphoenolpyruvate carboxykinase 1 | ENSSSCG00000007507 | F AGCCTGACCAAATCCACATC  R TGATGACCGTCTTGCTTTCA | 168 |
| miRNAs |  |  |  |  |
| miRNA | Assay ID | Mature miRNA sequence | |  |
| hsa-miR-26a-5p | 477995_mir | UUCAAGUAAUCCAGGAUAGGCU | |  |
| hsa-let-7a-5p | 478575_mir | UGAGGUAGUAGGUUGUAUAGUU | |  |
| hsa-mir-100-5p | 478224_mir | AACCCGUAGAUCCGAACUUGUG | |  |
| hsa-mir-378a-3p | 478349_mir | ACUGGACUUGGAGUCAGAAGGC | |  |
| hsa-mir-103a-3p | 478253_mir | AGCAGCAUUGUACAGGGCUAUGA | |  |
| hsa-miR-125b-5p | 477885_mir | UCCCUGAGACCCUAACUUGUGA | |  |
