# Supplementary material for: Identification of Molecular Mechanisms Related to Pig Fatness at the Transcriptome and miRNAome Levels
Source: Genes (Basel). 2020 May 29;11(6):600. doi: 10.3390/genes11060600 (PMC7348756; doi:10.3390/genes11060600)
Supplement: Supplementary file 1 [file genes-11-00600-s001.zip › Table S3.docx]

| Table S3. Tarbase experimentally supported interactions – the predicted targeted genes(N) for each od detected 14 miRNAs. | | |
| --- | --- | --- |
| mRNAs | Targeted genes | N |
| hsa-let-7a-5p | <http://snf-515788.vm.okeanos.grnet.gr/#mirnas=hsa-let-7a-5p&methods=Tarbase&selection=0> | 2163 |
| hsa-let-7c-5p | <http://snf-515788.vm.okeanos.grnet.gr/#mirnas=hsa-let-7c-5p&methods=Tarbase&selection=0> | 0 |
| hsa-let-7i-5p | <http://snf-515788.vm.okeanos.grnet.gr/#mirnas=hsa-let-7i-5p&methods=Tarbase&selection=0> | 1753 |
| hsa-miR-100-5p | <http://snf-515788.vm.okeanos.grnet.gr/#mirnas=hsa-miR-100-5p&methods=Tarbase&selection=0> | 393 |
| hsa-miR-10b-5p | <http://snf-515788.vm.okeanos.grnet.gr/#mirnas=hsa-miR-10b-5p&methods=Tarbase&selection=0> | 400 |
| hsa-miR-143-3p | <http://snf-515788.vm.okeanos.grnet.gr/#mirnas=hsa-miR-143-3p&methods=Tarbase&selection=0> | 684 |
| hsa-miR-24-3p | <http://snf-515788.vm.okeanos.grnet.gr/#mirnas=hsa-miR-24-3p&methods=Tarbase&selection=0> | 1771 |
| hsa-miR-26a-5p | <http://snf-515788.vm.okeanos.grnet.gr/#mirnas=hsa-miR-26a-5p&methods=Tarbase&selection=0> | 1540 |
| hsa-miR-378a-3p | <http://snf-515788.vm.okeanos.grnet.gr/#mirnas=hsa-miR-378a-3p&methods=Tarbase&selection=0> | 849 |
| hsa-miR-139-5p | <http://snf-515788.vm.okeanos.grnet.gr/#mirnas=hsa-miR-139-5p&methods=Tarbase&selection=0> | 459 |
| hsa-miR-142-5p | <http://snf-515788.vm.okeanos.grnet.gr/#mirnas=hsa-miR-142-5p&methods=Tarbase&selection=0> | 543 |
| hsa-miR-145-5p | <http://snf-515788.vm.okeanos.grnet.gr/#mirnas=hsa-miR-145-5p&methods=Tarbase&selection=0> | 490 |
| hsa-let-7d-5p | <http://snf-515788.vm.okeanos.grnet.gr/#mirnas=hsa-let-7d-5p&methods=Tarbase&selection=0> | 1596 |
|  |  |  |
|  |  |  |
