# Supplementary figures and images for: Identification of Molecular Mechanisms Related to Pig Fatness at the Transcriptome and miRNAome Levels
Source: Genes (Basel). 2020 May 29;11(6):600. doi: 10.3390/genes11060600 (PMC7348756; doi:10.3390/genes11060600)

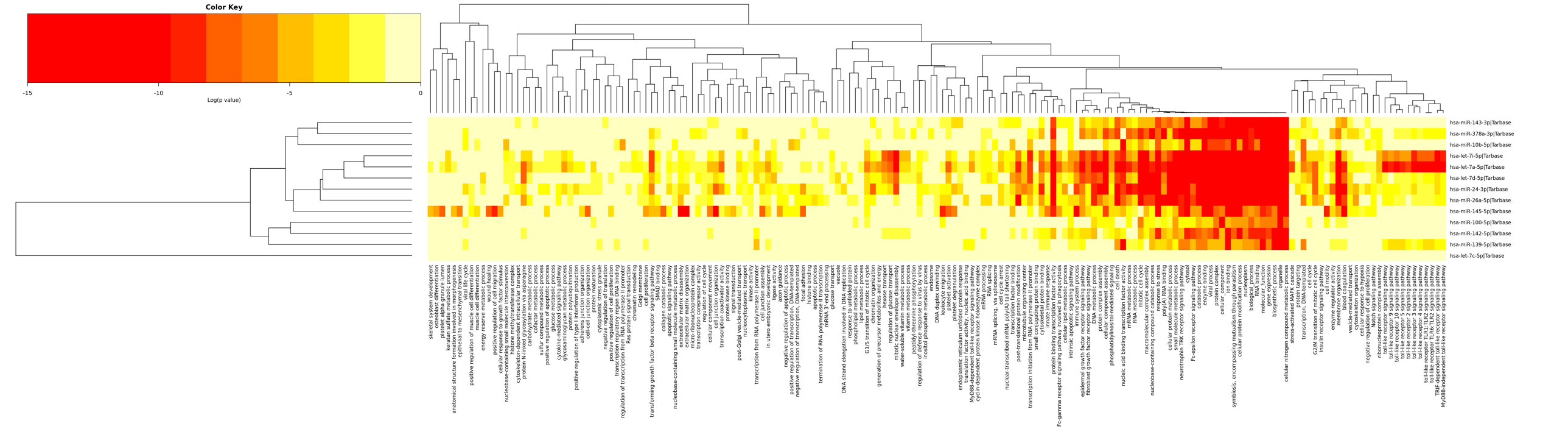

Supplement: Supplementary file 1 [file genes-11-00600-s001.zip › Figure S1.png]

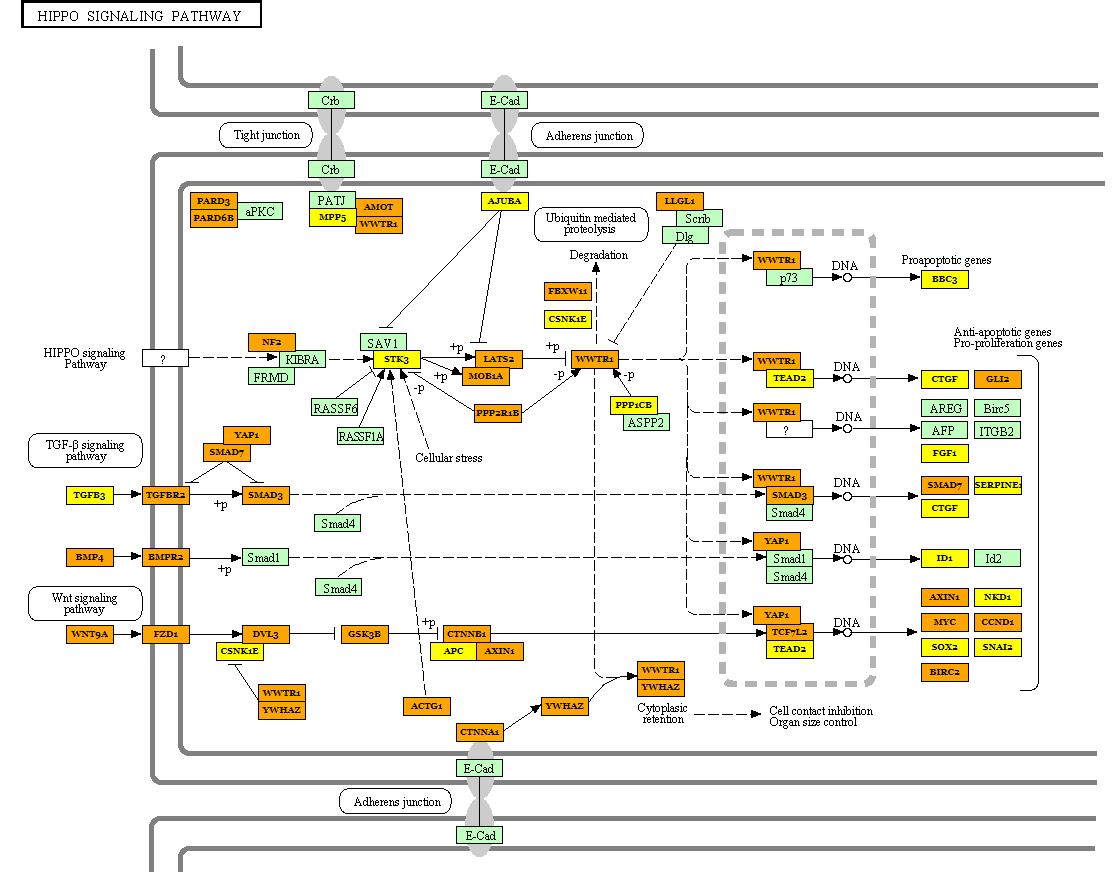

Supplement: Supplementary file 1 [file genes-11-00600-s001.zip › Figure S2.JPG]

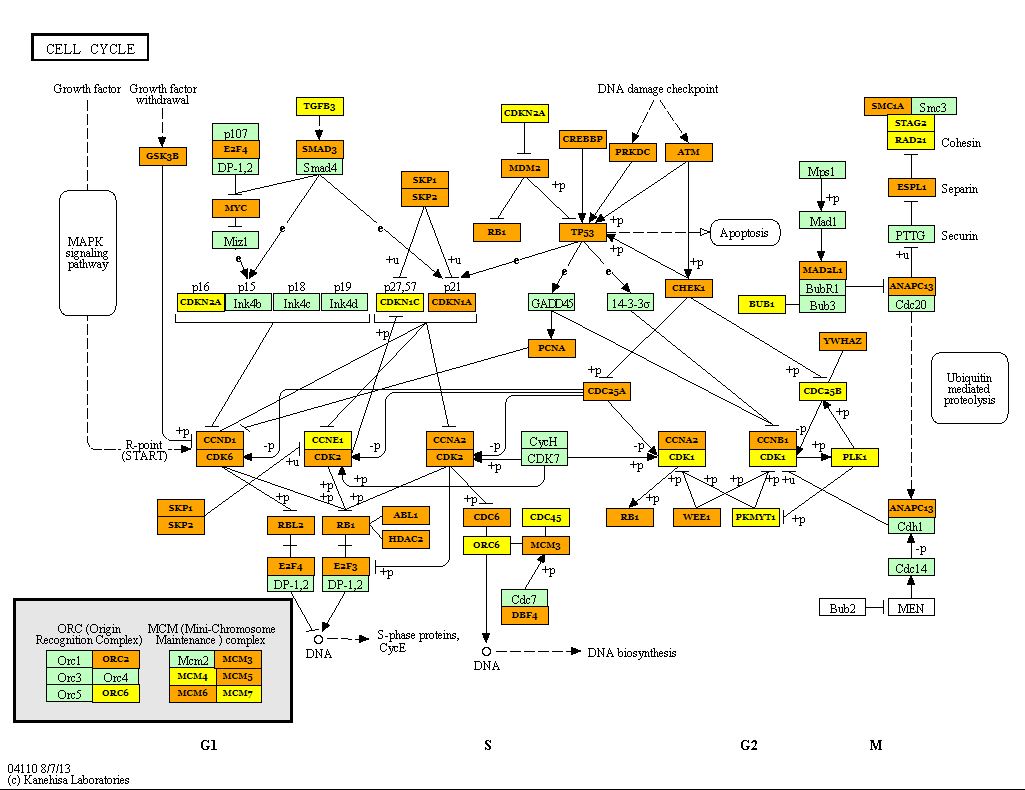

Supplement: Supplementary file 1 [file genes-11-00600-s001.zip › Figure S3.JPG]
